# MEG Node Degree for Focus Localization: Comparison with Invasive EEG

**DOI:** 10.3390/biomedicines11020438

**Published:** 2023-02-02

**Authors:** Stefan Rampp, Martin Kaltenhäuser, Nadia Müller-Voggel, Arnd Doerfler, Burkhard S. Kasper, Hajo M. Hamer, Sebastian Brandner, Michael Buchfelder

**Affiliations:** 1 Department of Neurosurgery, University Hospital Erlangen, 91054 Erlangen, Germany; 2 Department of Neurosurgery, University Hospital Halle (Saale), 06120 Halle (Saale), Germany; 3 Department of Neuroradiology, University Hospital Erlangen, 91054 Erlangen, Germany; 4 Epilepsy Center, Department of Neurology, University Hospital Erlangen, 91054 Erlangen, Germany

**Keywords:** MEG, epilepsy, epilepsy surgery, connectivity, graph theory, focus localization

## Abstract

Epilepsy surgery is a viable therapy option for patients with pharmacoresistant focal epilepsies. A prerequisite for postoperative seizure freedom is the localization of the epileptogenic zone, e.g., using electro- and magnetoencephalography (EEG/MEG). Evidence shows that resting state MEG contains subtle alterations, which may add information to the workup of epilepsy surgery. Here, we investigate node degree (ND), a graph-theoretical parameter of functional connectivity, in relation to the seizure onset zone (SOZ) determined by invasive EEG (iEEG) in a consecutive series of 50 adult patients. Resting state data were subjected to whole brain, all-to-all connectivity analysis using the imaginary part of coherence. Graphs were described using parcellated ND. SOZ localization was investigated on a lobar and sublobar level. On a lobar level, all frequency bands except alpha showed significantly higher maximal ND (mND) values inside the SOZ compared to outside (ratios 1.11–1.20, alpha 1.02). Area-under-the-curve (AUC) was 0.67–0.78 for all expected alpha (0.44, ns). On a sublobar level, mND inside the SOZ was higher for all frequency bands (1.13–1.38, AUC 0.58–0.78) except gamma (1.02). MEG ND is significantly related to SOZ in delta, theta and beta bands. ND may provide new localization tools for presurgical evaluation of epilepsy surgery.

## 1. Introduction

More than 50 million people worldwide suffer from epilepsy. While a majority can be treated adequately with anti-seizure medication (ASM), aproximately one third of patients are pharmacoresistant [1]. In these patients, epilepsy surgery is a viable therapy option with seizure freedom rates of approximately 60% one year after surgery; however, this depends on the etiology [2]. An essential prerequisite for succesful epilepsy surgery is the exact localization of the epileptogenic zone [3]. To this end, a wide spectrum of diagnostic methods is employed during presurgical evaluation of epilepsy, ranging from MRI (magnetic resonance imaging), SPECT (single-photon emission computed tomography) and PET (positron emission tomography) to neuropsychological evaluation, magnetoencephalography (MEG) and surface/invasive electroencephalography (EEG). The latter play an especially important role as they directly acquire neurophysiologic epileptic activity. EEG/MEG data is utilized for focus localization, either by classic visual interpretation or using methods of source imaging. Numerous studies have demonstrated that taking results of electric or magnetic source imaging (ESI/MSI) into account in planning invasive recordings and epilepsy surgery is associated with significantly better postoperative seizure control in the short- and long-term [4,5,6,7,8]. This is true especially in difficult cases, such as patients without a clear structural lesion on MRI, as source imaging may provide crucial information enabling a patient to proceed to surgery [9,10]. However, the current clinical practice of ESI and MSI utilizes ictal rhythms and interictal epileptic discharge (IED) patterns. Consequently, if no or too few patterns are available, source imaging cannot be applied.

In recent years, a growing body of evidence has described electrophysiological changes associated with epileptic networks which go beyond the classical markers. Increases of oscillatory activity in MEG and invasive and scalp EEG in different frequency bands have been demonstrated with varying specificity for areas exhibiting conventional ictal and interictal patterns [11,12,13,14]. A further category of such surrogate markers of the epileptic network above and beyond activation strength are measures of connectivity, i.e., the communication structure between different areas.

The common underlying hypothesis of these approaches is that the epileptic focus’ spontaneous activity is communicated to neighboring or even distant areas via pre-existing physiological or newly formed pathological pathways. This communication then manifests as either classical pattern propagation or as more subtle influences. The epileptic focus could then be identified within this functional network as a hub node by analyzing continuous resting state data [15].

In a previous study [15], we investigated this hypothesis by employing the “node degree” measure, a graph-theoretical parameter which describes the communicative involvement of network nodes and exhibits especially high values within network hubs. We were able to demonstrate that patients with pharmacoresistant focal epilepsies show significantly higher overall node degree values compared with healthy controls, independent of several practically relevant recording conditions. However, since we selected a representative series of consecutive patients from presurgical evaluation, irrespective of etiology and whether or not invasive EEG and epilepsy surgery had been conducted, we did not evaluate spatial distribution of network hubs in detail.

Consequently, we now focus on the localization of such network hubs. The rationale of the current study is to explore MEG node degree in different frequency bands in a consecutive series of patients who have undergone invasive EEG recordings (iEEG). We compare the ability of the node degree parameter to localize the seizure onset zone (SOZ) in invasive EEG, which we utilize as reference standard. A high localization performance would support the clinical value of utilizing this marker in addition or alternatively to conventional IED-based source imaging for epileptic focus localization.

## 2. Materials and Methods

### 2.1. Participants

A total of 50 consecutive adult patients with pharmacoresistant epilepsies were selected retrospectively who had undergone presurgical evaluation for epilepsy surgery between 11/2012 and 01/2019. Further inclusion criteria were invasive EEG evaluation and a routine MEG recording as part of the presurgical workup. Patients were excluded if the iEEG could not determine the seizure onset zone or if routine MEG analysis could not be performed due to poor data quality. Outcome after epilepsy surgery was not the primary endpoint of our study; consequently, specific outcomes or a minimum duration of postoperative follow-up were not required for inclusion. Due to the retrospective nature, requirements and results of the presented analyses did not influence clinical decision making regarding the iEEG-recordings or viability and extent of surgery.

The procedures of this study were positively reviewed by the Institutional Review Board of the medical faculty, University Erlangen-Nuremberg, Germany (registration number 179_19 B, 13.6.2019). Patients provided their written informed consent for the use of their data in retrospective research studies.

### 2.2. MEG Acquisition

MEG data were recorded with a 248-channel whole head system (Magnes 3600 WH, 4D-Neuroimaging, San Diego, CA, USA) in a magnetically shielded room (Vacuumschmelze GmbH, Hanau, Germany). Recordings were part of clinical presurgical evaluation and followed an in-house protocol, consisting of one run of 20 min with a sampling rate of 508.6 Hz and two 10-min runs with 2034.5 Hz. Analog bandpass filters were used with 0.1 or 1 Hz high-pass frequency and 200 Hz or 800 Hz lowpass depending on sampling rate. Patients were recorded in a supine position with eyes closed. For the purposes of the presented study, only 10 min of the 20 min-run were selected for analysis to limit computational demands.

Prior to data acquisition, head position was acquired using five coils attached close to the left and right preauricular points, the nasion, vertex and inion. Coil positions and headshape for coregistration with MRI were recorded using a 3D digitizing pen (Polhemus, Colchester, VT, USA).

### 2.3. MRI Acquisition

All patients underwent MRI scanning with a high-resolution 1.5 or 3 T MRI system (Magnetom Trio and Aera, Siemens AG, Erlangen, Germany) using an optimized protocol for evaluation of patients with epilepsy, including a T1-weighted isotropic 1 mm 3D MP-RAGE (magnetization prepared rapid gradient echo) sequence which was used for construction of the volume conduction model and visualization of the results.

### 2.4. Presurgical Evaluation

Presurgical evaluation consisted of a non-invasive phase 1 evaluation, which included video-EEG, MRI, MEG, and neuropsychological evaluation, as well as PET and/or SPECT when clinically indicated. All data were reviewed in a multidisciplinary case management conference, which decided on the need for further diagnostic evaluation or the viability of epilepsy surgery. Due to the inclusion criteria, all patients proceeded to invasive phase 2 evaluation. Invasive EEG over several days was recorded using subdural and/or depth electrodes (Ad-Tech, Medical Instruments Corporation, Racine, WI, USA). Placement of electrodes was planned according to the consensus of phase 1 evaluation. A second patient management conference then decided on viability and extent of surgery.

Results from invasive EEG monitoring served as the reference standard in the current study. Involvement of individual iEEG contacts in the seizure onset were taken from clinical documentation. Locations of electrodes were determined visually using post-implantation MRI and CT (computed tomography) acquired during presurgical evaluation and were related to individual lobes and regions using the automatic anatomical labeling (AAL) atlas [16].

If available, data on performed epilepsy surgery and postoperative outcomes were collected from clinical documentation.

### 2.5. MEG Analysis

Preprocessing and analysis steps correspond to our previous study on MEG node degree in patients with epilepsy [15]. Here we provide an overview of the procedure.

All processing steps were conducted using the fieldtrip software version 20180124 (https://www.fieldtriptoopbox.org, accessed on 24 January 2018) [17] and SPM8 (https://www.fil.ion.ucl.ac.uk/spm/, accessed on 24 January 2018) on Matlab R2020b (The Mathworks, Natick, MA, USA).

### 2.6. Preprocessing

The individual patients’ 3D T1 MRI datasets were used to segment brain and skin compartments. Coregistration with the MEG coordinate system was achieved by matching the head shape-points acquired during the MEG acquisition to the segmented skin surface. A single-shell volume conductor model [18] was set up using the segmented brain compartment.

For the definition of source space nodes, an MNI (Montreal Neurological Institute)-template grid with 10 mm resolution was fit to the brain compartment (total of 3294 nodes). This was performed to allow for inter-subject comparability as well as comparison of node degree values with the AAL atlas.

As a first step of MEG preprocessing, the 10 min of data were divided into 2-s epochs. Channels and epochs with strong artifact contamination on visual inspection were excluded from further analysis. Epochs were then subjected to an independent component analysis (ICA) using the “runica” algorithm [19]. ICA components corresponding to eye blinks, eye movement and electrocardiography (ECG) artifacts were visually identified and removed. Interictal epileptic discharges were left in the data; seizures did not occur during the MEG recordings.

### 2.7. Source Analysis

Preprocessed data were then subjected to a multitaper frequency transformation in 1 Hz steps in delta (1–4 Hz), theta (4–7 Hz), alpha (8–15 Hz), beta (15–30 Hz) and low gamma (30–45 Hz) bands. The results were subjected to source analysis using a partial conical coherence beamformer with 5% regularization. Source-reconstructed data was then projected to the dominant orientation.

### 2.8. Connectivity and Graph Analysis

Imaginary part of coherence [20] was calculated as connectivity measure between all pairs of source space nodes. The resulting values were thresholded at the 95th percentile to construct a corresponding graph. The graph-theoretical node degree (ND) measure was then calculated for each source space node.

For comparison with iEEG, node degrees were parcellated based on the AAL atlas, which was also used for the description of ictal iEEG findings. For each parcel, i.e., AAL region, the maximal node degree value (mND) was determined as this had shown a consistent contrast to healthy controls in the previous study [15].

### 2.9. Statistical Evaluation

The aim of the statistical evaluation was to test how well node degree values can localize areas that show the seizure onset or early propagation in iEEG. This question was addressed on a lobar level (left/right frontal, temporal, parietal, occipital, and insula), as well as on a sublobar level in terms of AAL defined regions, but excluding subcortical structures and the cerebellum. In the previous study [15], all investigated frequency bands showed a contrast between patients and controls. Since quantitative differences were small, the current study did not have a specific hypothesis on which frequency band would yield the best localization results. Consequently, we investigated their value in an exploratory manner; however, we also corrected for multiple comparisons by false-discovery-rate (FDR) adjustment [21].

Comparisons were performed by comparing node degree maxima (mND) between lobar and sublobar SOZ (seizure onset) and non-SOZ regions using the paired Wilcoxon signed-rank test. Furthermore, area-under-the-curve (AUC) values of receiver-operator statistics (ROC) were calculated, taking node degree values as score and the list of areas showing epileptic activity in iEEG vs. those that do not as target (yes/no) labels. Statistical significance of AUC values was evaluated using a bootstrap procedure. To this end, node degree values were shuffled with respect to the iEEG activity labels before calculating AUC for this surrogate data. This procedure was then repeated 1000 times. Comparison of the actual AUC to the resulting distribution then yielded a *p*-value. The conventional value of 0.05 was applied as the threshold for statistical significance.

Further exploratory analyses were conducted, but these were limited to the sublobar level for brevity. Inside:outside SOZ ratios were correlated to the global node degree maximum to evaluate a potential marker for the quality of node degree analysis. To evaluate the relationship to IED detection in routine MEG analysis, AUC values between patients with and without such findings were compared with the Wilcoxon rank-sum test. AUC values were also compared between patients with an Engel 1 outcome vs. those with Engel 2 or 3 (Engel 4 did not occur) using the rank-sum test.

## 3. Results

### 3.1. Patients

A total of 68 consecutive patients were screened to select 50 patients according to the inclusion/exclusion criteria. Among the 50 patients, 27 were female (54%). Mean age was 31 years (±9.7, range 18–53 years). Table 1 provides an overview of patients’ characteristics.

### 3.2. Presurgical Evaluation

MRI did not show a lesion in 14 patients (28%).

Invasive EEG showed the seizure onset within the left hemisphere in 25 (50%) patients and in 24 (48%) patients on the right, and separate left- and right-sided SOZ in one patient (2%). The SOZ was in the temporal lobe in 14 (28%), frontal lobe in 24 (48%), parietal lobe in 9 (18%), occipital lobe in 2 (4%) and insula in 3 (6%) patients.

Interictal epileptic discharges were not detected in the MEG data in 12 patients (24%). MEG findings were concordant with the iEEG SOZ on a lobar level in 25 (66% of 38 patients with IEDs in MEG), overlapping in 9 (24%) and not concordant in 4 (10%).

A total of 28 patients underwent epilepsy surgery; the outcome was Engel 1A in 17 (61% of 28 operated patients), 1B in 1 (4%), 2D in 1 (4%) and 3A in 9 (31%) after a median follow-up of 24 months (range 3–72 months, 21 patients with at least 12 months).

### 3.3. Node Degree

**Figure 1:**
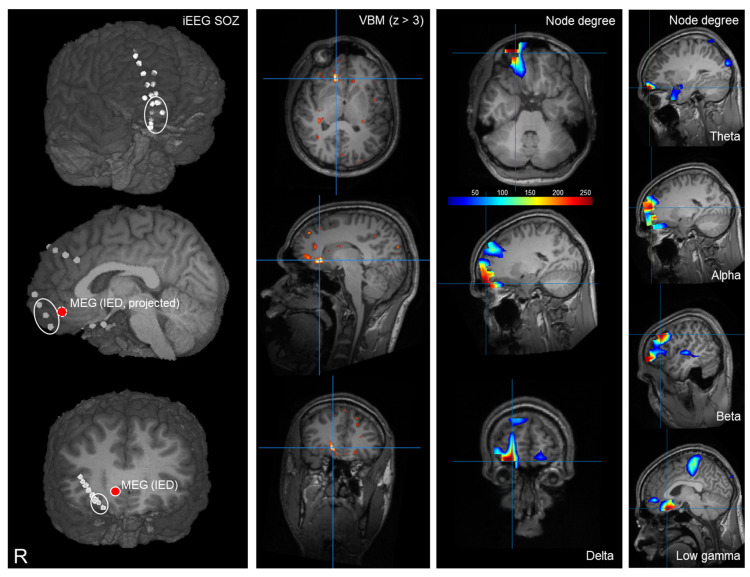
Example case of a patient with right fronto-polar focal cortical dysplasia type IIb. The first column shows the iEEG findings; fronto-polar SOZ electrodes are circled. Lower inset shows depth electrodes inserted into the lesion. The circle marker shows the localization from routine MEG analysis based on IEDs. Second column shows MRI postprocessing (voxel-based morphometry, MAP19, combined z-score, thresholded at z > 3) superimposed on the patient’s T1. Third column shows delta band node degree (thresholded at 95th percentile to illustrate maximum, see Supplementary Figure S1 for full distributions). Right-most column shows sagittal slices of node degree in the other frequency bands (thresholded). The patient underwent epilepsy surgery and was seizure-free thereafter for several years; however, the patient then developed recurrent seizures with a reduced frequency.

**Figure 2:**
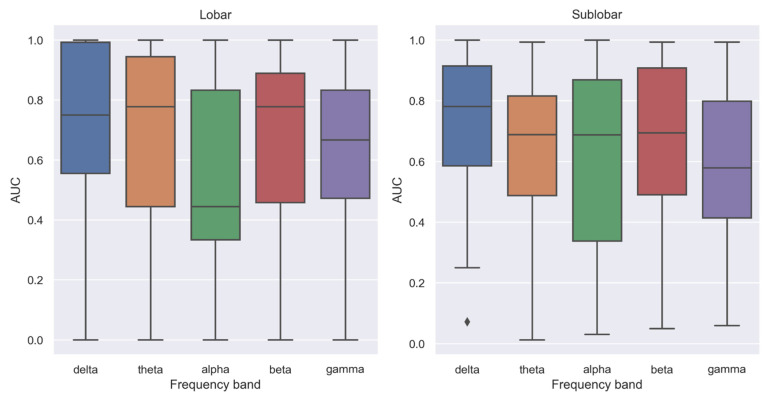
Overview of localization performance of node degree in different frequency bands for the seizure onset in invasive EEG on a lobar and sublobar (AAL atlas region) level.

Figure 1 and Supplementary Figure S1 show an example of node degree distribution and relationship to the seizure onset in invasive EEG. Table 2, Table 3 and Table 4 as well as Figure 2 provide further details for the summary given here.

### 3.4. Lobar Level

On a lobar level, all frequency bands except alpha showed significantly higher mND values inside the SOZ compared to outside (Table 2).

Node degree showed median AUC values of 0.75 in the delta band, 0.78 in the theta, 0.44 in the alpha, 0.78 in the beta and 0.67 in the low gamma band (Figure 2). Except for alpha, localization performance in all frequency bands were statistically significant (Table 3). Differences between frequency bands were significant only for alpha with delta, theta and beta bands (Table 4). Comparison of alpha and low gamma yielded a *p*-value slightly above the chosen threshold for significance before FDR-adjustment (*p* = 0.051 unadjusted, *p* = 0.13 FDR-adjusted).

In a total of 7 patients, AUC values for all frequency bands exceeded the arbitrary threshold of 0.7. Excluding alpha and gamma due to lowest overall performances yields 12 patients with such high AUC values in all other bands.

### 3.5. Sublobar Level

On a sublobar level using AAL regions [16], ratios of inside:outside also indicated significantly higher mND inside the iEEG SOZ for all frequency bands except gamma (Table 2). This ratio was partially correlated to the global maximal node degree value: delta 0.29 (*p* = 0.054, FDR-adjusted), theta 0.27 (*p* = 0.058), alpha 0.43 (*p* = 0.003), beta 0.43 (*p* = 0.003) and gamma 0.62 (*p* < 0.001).

Node degree showed median AUC values of 0.78 in the delta band, 0.69 in the theta, alpha and beta band, and 0.58 in the low gamma band. All AUC values reached statistical significance (Table 3). Comparing AUC values between frequency bands on a sublobar level yielded statistically significant differences only for delta—low gamma and beta— low gamma and only before correcting for multiple comparisons (Table 4).

On the sublobar level, five patients had AUC values > 0.7 in all bands and nine when alpha and gamma are excluded. AUC values between patients with and without IEDs in routine MEG evaluation did not differ significantly (theta *p* = 0.09, *p* > 0.1 in the remainder, not FDR-adjusted). AUC did not show significant differences comparing patients with and without Engel 1 at least 12 months after surgery (13 vs. 8 patients).

## 4. Discussion

In the current study, we evaluated the localization performance of MEG node degree using the seizure onset in invasive EEG as the reference standard. The results show significant increases inside vs. outside the seizure onset zone in iEEG, as well as a good localization performance, especially in the delta, theta and beta band. In contrast, node degree in the low gamma and especially alpha band showed high variability and limited localization performance.

In our previous study [15], we demonstrated that node degree is consistently higher in patients with focal epilepsy compared to healthy controls in all frequency bands (delta, theta, alpha, beta and low gamma), largely independent of recording conditions. We had not, however, evaluated the spatial distribution of node degree or compared it with a reference standard. The current results now show that the increase is significantly related to the seizure onset zone as determined by iEEG, with higher node degree values inside the SOZ yielding good localization performance.

### 4.1. Connectivity Alterations in Epilepsy

Such increases in connectivity measures have been reported by other authors, as summarized in a recent review by Xu et al. [22]. The available evidence highlights group differences of connectivity in extended networks in patients with focal vs. generalized epilepsies [23,24] and compared with healthy controls [25,26].

Changes in the vicinity of the putative epileptogenic zone, and thus potentially useful for epileptic focus localization, are reported by fewer authors. Krishan et al. [27] were able to localize the epileptic focus in a small group of five patients based on resting state connectivity irrespective of the presence of IEDs in the data using generalized partial directed coherence. Aydin et al. [28] found more isolated resting state networks in patients who became seizure-free after epilepsy surgery compared with patients with persisting seizures using amplitude envelope correlation. Similarly, Ramaraju et al. [29] report a larger portion of network hubs (determined by amplitude correlation) contained in the resection in seizure-free patients after surgery. Although Englot et al. [30] report reduced overall connectivity in comparison with healthy controls—contrasting findings of most studies in the field [22]—they also found increases in the resection area of successfully operated patients using the imaginary part of coherence.

Further studies utilize connectivity measures to investigate IEDs [31], epileptic high frequency oscillations (HFO) [32] and ictal rhythms [33], largely with the goal to improve the accuracy of focus localization above and beyond more conventional methods.

In the context of our study, the recent publication by Fujiwara et al. on IED connectivity [31] seems especially relevant as they use a very similar approach, in contrast to the broad methodological spectrum of the previously mentioned studies. Fujiwara et al. [31] investigated 31 patients using MEG eigencentrality of weighted phase-lag-index connectivity, a graph-theoretical parameter that describes the importance of an individual node within a network, similar to the node degree used in our study. They showed that the localization of primary hubs was closer to the iEEG SOZ compared with conventional dipole localization. In addition, complete resection of either dipoles or primary nodes were related to better surgical outcomes. In contrast to our study, they investigated only short data epochs around IEDs, while our analysis evaluated continuous resting-state data irrespective of occurrence or positions of IEDs.

Despite the methodological heterogeneity, current evidence points to a functional role of network dynamics in epilepsy with promising potential for clinical application.

### 4.2. Differences between Frequency Bands

In our study, node degree in delta, theta and beta bands showed the highest association with the SOZ. In contrast, the performance of node degree in alpha and gamma bands was more limited. The ratio of alpha mND inside vs. outside the SOZ lobe did not significantly differ from an equal distribution and showed very high variability of AUC values on both lobar and sublobar levels. While gamma mND also showed low AUC and inside:outside ratio values, these limitations were much more consistent than in the alpha band. Consequently, we assume different reasons for the low performance.

A problem for the gamma node degree is certainly the low signal-to-noise ratio (SNR) of fast MEG/EEG activity [34]. In addition, generators of gamma activity are thought to be spatially constrained and limited to smaller areas. Furthermore, gamma oscillations often occur in short bursts and less as continuous oscillations over longer periods [35]. Consequently, gamma band connectivity may be restricted to shorter time segments with increased spatial variability and lower signal amplitude. In comparison with the lower frequency bands, detection of gamma activity as well as its connectivity structure may thus be technically more difficult, resulting in the lower performance.

However, the same reasoning does not seem to be suited for explaining the limited alpha band results. According to the 1/f power-law [34], alpha displays overall better SNR than beta. In addition, beta band activity is also thought to occur in bursts [36] rather than ongoing oscillations, as is the case for alpha. Despite these characteristics, beta performance was overall better than performance of the alpha band.

Instead, the putative functional role of alpha in physiologic networks may provide a tentative explanation. At least in the context of attentional processes, alpha is associated with inhibition of information that is irrelevant to a given task, which it achieves by masking out respective networks [37]. Details of how this masking function is coordinated remain largely unresolved. A putative direct low-level influence of task-relevant activity in addition to top-down modulation suggests a mechanism by which spontaneous epileptic activity may induce alpha oscillations. Reminiscent of lateral inhibition [38], task processes may inhibit connected networks involved with unrelated or concurrent parallel processing to improve performance. Epileptic activation could then take the role of such task-related activity, i.e., epileptic activity would be “mistaken” as physiological processing. The functional architecture would then induce alpha activity in connected networks as a response to such erroneous epileptic “task processing”. While alpha increases could then occur in the vicinity of the SOZ, pathways to distant regions could lead to more widespread activation. Due to the induction by the common driver of the epileptic focus, the resulting alpha oscillations could show synchronization and functional connectivity. Such widespread connectivity is reflected in our data by the lower localization performance at the lobar level. This contrasts with the equal or better performance of other frequency bands, which likely benefit from the coarser resolution of the lobar vs. sublobar level.

The good performance of node degree in delta, theta and beta bands corresponds well to evidence showing that the quantity of resting state oscillations in these frequency bands may be useful for epileptic focus localization [11,12,14,39,40]. Such local power increases could then reflect another perspective on activated networks. Imaginary part of coherence measures phase relations [20], and thus it seems conceivable that an activated network, if it is not constrained to few, focally concentrated nodes, shows both power-and connectivity-increases. Connectivity (and its description by graph-theory) may thus provide complementary information to power analysis, although we have not investigated this in the current study.

### 4.3. Clinical Aspects

Node degree analysis in epileptic focus localization has several interesting aspects for clinical application. Conventional MEG/EEG source imaging relies on subjective identification of IEDs [4,41], potentially supported by automated procedures [42]. Except for artifact rejection, our approach is completely automated and based on statistical properties of the data; it is thus reproducible and objective, albeit dependent on technical details, such as the chosen inverse solution and connectivity measure and their parameters. In terms of practical application, automation avoids the considerable time otherwise needed for visual IED detection and localization.

The unsupervised evaluation of resting state data, however, also results in a crucial downside. Any data, and specifically also physiologic data from healthy controls, yields node degree distributions with local maxima [15]. Correspondingly, the specificity of local node degree increases may be limited and, to some degree, unknown. In contrast, conventional or connectivity-based analysis of IEDs does not suffer from this issue due to the clear association to epilepsy, albeit by subjective interpretation. A potential solution, although only partial at this point, is to evaluate the levels of node degree values. Our previous study [15] showed that focal epilepsy leads to overall higher node degree levels compared with healthy participants. In concordance with this finding, the observed maximum per patient and frequency band correlated with the ratio of node degree inside to outside the SOZ in the present data. In practice, this means that overall low node degree values suggest unreliable localization results, although correlations were far from perfect. Ideally, values exceeding “normal” levels should be determined by regional comparison with healthy controls. Such an approach may also contribute to improved localization accuracy.

The observation of cases with low node degree values and incorrect localizations, i.e., “node degree negative” cases, is hardly surprising. In conventional MEG focus localization, a rate of ~20–30% of patients without IEDs is expected [4]. A main reason for this is the limited recording time of about one hour. In the present study, we even only analyzed 10 min due to computational constraints. Although this duration still exceeds recordings of other successful studies [28,29,30], it seems reasonable to assume that a portion of the recordings—not necessarily the same as the datasets without IEDs—contain little or no epilepsy-associated node degree increases. Correspondingly, analysis of longer data may offer further potential improvement.

Patients with an Engel 1 outcome after epilepsy surgery did not show significantly higher AUC values than those who did not. Other authors, however, have shown that resection of prominent network nodes is favorable with regard to postsurgical seizure control [29,30,31]. There are several potential reasons for this difference. First, AUC values referred to SOZ in iEEG. Investigation of the relationship to the resection volume in operated patients was not our primary goal, as we were interested in a comparison with the de-facto gold-standard of epileptic activity, independent of further influences such as viability of surgery, extent of resection, vicinity of essential functional cortex, postsurgical medication, and compliance, etc. Correspondingly, our selection criteria were not chosen to include a large and representative sample of surgical procedures, resulting in only 21 operated patients with a minimum follow-up of one year. Finally, the relation to outcome is likely also impacted by the “node degree negative” cases, in whom the connectivity likely depicts physiological structures unrelated to the focus and surgical outcome.

### 4.4. Limitations

A further limitation of our study is the fact that we did not exclude epochs with IEDs in MEG from the analysis. Strictly speaking, we therefore cannot tell whether the observed connectivity is mainly caused by such patterns or by subtle alterations of background activity. However, since we included considerably longer data than other studies [28,29,30], the overall contribution of IEDs to the recording duration is minute in basically all patients. Furthermore, the comparison of patients with and without IEDs in MEG did not reveal any statistically significant differences.

We also only made comparisons with the SOZ in iEEG and did not extend the analysis to the irritative zone. While such investigations in future studies could provide further insights into the underlying pathophysiology of epileptic networks, the clinically most relevant aspect is the localization of the SOZ as a surrogate marker of the epileptogenic zone [3]—our main interest in the current study.

Finally, the study design is a retrospective one, with the typical limitations regarding bias, representativeness of the sample, etc. However, by selecting a consecutive series of patients from clinical routine and by using automated procedures, we hope to have avoided most of the common pitfalls. Unavoidable, however, was the heterogeneity of anti-seizure and other medication. MEG recordings at our institution are usually conducted before or after video-EEG monitoring, i.e., ASM were not tapered and—again due to the retrospective nature—not limited to certain drugs.

## 5. Conclusions

Increases in MEG node degree from resting state data are significantly related to the seizure onset zone determined by invasive EEG at a lobar and sublobar level. This relationship is especially apparent for the delta, theta and beta bands. In contrast, alpha and gamma node degrees show overall limited association with the seizure onset zone. Additionally, alpha node degree shows a diffuse distribution and considerable variability. Node degree may provide new focus localization tools for presurgical evaluation of epilepsy surgery; however, this requires further optimization before implementation in clinical practice.

## Figures and Tables

**Table 1:** Patient characteristics. FCD—Focal cortical dysplasia, HS—hippocampal sclerosis, PMG—polymicrogyria. Patients with no lesion on MRI are included in the “unclear” etiology category.

Number	Sex	Age at MEG	iEEG SOZ		Etiology	Epilepsy Surgery Outcome	Follow-Up
			Side	Lobe		(Engel)	(Months)
1	F	21	L	parietal	FCD	1A	48
2	M	31	R	occipital	Unclear lesion	1A	72
3	F	20	R	frontal	Unclear		
4	M	18	L	temporal	FCD		
5	F	28	L	frontal	FCD	3A	24
6	F	29	L	frontal	FCD		
7	F	29	L	frontal	FCD	1A	29
8	F	26	R	parietal	Unclear	3A	24
9	M	43	R	frontal	FCD	1A	43
10	F	52	L	frontal	FCD		
11	F	44	L	temporal	HS		
12	F	50	L	temporal	Unclear		
13	M	53	R	temporal	Unclear	1A	6
14	F	24	L	temporal	HS	1A	36
15	M	18	R	frontal	FCD	3A	33
16	F	42	R	frontal	FCD	1A	36
17	M	24	R	frontal	FCD		
18	M	26	R	parietal	Unclear		
19	M	36	L	temporal	Unclear	3A	36
20	F	23	R	frontal	FCD	3A	12
21	M	21	L	insel	Unclear		
22	F	34	R	frontal	Unclear		
23	M	28	L	frontal	Unclear	2D	24
24	M	20	R	frontal	Unclear		
25	F	29	L	parietal	Unclear		
26	F	30	L	temporal	HS	1A	24
27	M	27	L	frontal	MCD	1A	24
28	M	28	L	frontal	FCD	1B	6
29	F	42			Unclear	1A	24
30	M	33	R	temporal	Unclear	1A	12
31	M	20	R	parietal	Unclear	1A	6
32	F	48	R	frontal	FCD	3A	6
33	F	36	L	frontal	FCD	1A	34
34	M	26	L	frontal	FCD	3A	30
35	M	47	R	frontal	Periv. heterotopia.		
36	M	42	L	insula	FCD		
37	F	19	L	parietal	Postischemic		
38	M	22	R	parietal	Unclear		
39	M	22	R	frontal	FCD	1A	6
40	F	19	R	frontal	Unclear	1A	12
41	M	32	L	frontal	PMG	3A	12
42	F	26	R	frontal	Unclear		
43	F	48	L	temporal	Unclear	1A	18
44	F	24	L	occipital	Unclear	3A	3
45	F	33	L	parietal	FCD	1A	3
46	F	33	R	temporal	HS		
47	M	32	R	frontal	FCD		
48	F	26	R	temporal	Unclear		
49	F	33	L	temporal	Unclear		
			R	parietal			
50	M	33	L	insula	Unclear		

**Table 2:** Ratio of node degree values inside vs. outside the iEEG SOZ. Significance levels: * *p* < 0.05, ** *p* < 0.01, *** *p* < 0.001.

Frequency Band	Inside:Outside	1st–3rd Quartile	*p* (Uncorrected)	*p* (FDR-Adjusted)	Adjusted Significance Level
**Lobar**					
Delta	1.19	0.98–1.55	<0.0001	<0.0001	***
Theta	1.14	0.97–1.48	<0.001	<0.001	***
Alpha	1.02	0.85–1.28	0.28	0.28	
Beta	1.20	0.96–1.45	<0.001	<0.001	***
Low gamma	1.11	0.93–1.26	0.016	0.02	*
**Sublobar (AAL)**					
Delta	1.38	1.07–1.64	<0.0001	<0.0001	***
Theta	1.22	0.99–1.44	<0.0005	<0.001	***
Alpha	1.36	0.44–2.59	0.009	0.011	*
Beta	1.13	0.92–1.63	0.001	0.002	**
Low gamma	1.02	0.84–1.30	0.16	0.16	

**Table 3:** Localization performance of node degree values for invasive EEG seizure onset. Significance levels: * *p* < 0.05, *** *p* < 0.001.

Frequency Band	Median AUC	1st–3rd Quartile	*p* (Uncorrected)	*p* (FDR-Adjusted)	Adjusted Significance Level
**Lobar**					
Delta	0.75	0.56–1.0	<0.0001	<0.0001	***
Theta	0.78	0.44–0.94	<0.0005	<0.001	***
Alpha	0.44	0.33–0.83	0.23	0.23	
Beta	0.78	0.44–0.89	<0.0001	<0.0001	***
Low gamma	0.67	0.44–0.83	<0.001	<0.001	***
**Sublobar (AAL)**					
Delta	0.78	0.58–0.92	<0.0001	<0.0001	***
Theta	0.69	0.47–0.82	<0.0001	<0.0001	***
Alpha	0.69	0.33–0.87	0.009	0.011	*
Beta	0.69	0.49–0.91	<0.0002	<0.0002	***
Low gamma	0.58	0.41–0.80	0.037	0.037	*

**Table 4:** Comparison of frequency band localization performance of node degree values for invasive EEG seizure onset. Significance levels: * *p* < 0.05, *** *p* < 0.001.

Comparison	*p* (Uncorrected)	*p* (FDR-Adjusted)	Adjusted Significance Level
**Lobar**			
Delta—Theta	0.25	0.36	
Delta—Alpha	0.003	0.015	*
Delta—Beta	0.48	0.59	
Delta—Low gamma	0.088	0.18	
Theta—Alpha	0.009	0.03	*
Theta—Beta	0.56	0.62	
Theta—Low gamma	0.63	0.63	
Alpha—Beta	<0.001	0.006	***
Alpha—Low Gamma	0.051	0.13	
Beta—Low Gamma	0.25	0.36	
**Sublobar (AAL)**			
Delta—Theta	0.18	0.35	
Delta—Alpha	0.07	0.25	
Delta—Beta	0.22	0.37	
Delta—Low gamma	0.01	0.12	
Theta—Alpha	0.41	0.46	
Theta—Beta	0.92	0.92	
Theta—Low gamma	0.10	0.26	
Alpha—Beta	0.36	0.45	
Alpha—Low Gamma	0.35	0.45	
Beta—Low Gamma	0.04	0.22	

## Data Availability

Data is available from the authors upon reasonable request.

## Supplementary Materials

The following supporting information can be downloaded at: https://www.mdpi.com/article/10.3390/biomedicines11020438/s1, Figure S1: Full node degree distribution of case shown in Figure 1.

## References

[1] Behr C., Goltzene M.A., Kosmalski G., Hirsch E., Ryvlin P. (2016). Epidemiology of epilepsy. Rev. Neurol..

[2] Blumcke I., Spreafico R., Haaker G., Coras R., Kobow K., Bien C.G., Pfafflin M., Elger C., Widman G., Schramm J. (2017). Histopathological Findings in Brain Tissue Obtained during Epilepsy Surgery. N. Engl. J. Med..

[3] Rosenow F., Luders H. (2001). Presurgical evaluation of epilepsy. Brain.

[4] Rampp S., Stefan H., Wu X., Kaltenhauser M., Maess B., Schmitt F.C., Wolters C.H., Hamer H., Kasper B.S., Schwab S. (2019). Magnetoencephalography for epileptic focus localization in a series of 1000 cases. Brain.

[5] van Mierlo P., Vorderwulbecke B.J., Staljanssens W., Seeck M., Vulliemoz S. (2020). Ictal EEG source localization in focal epilepsy: Review and future perspectives. Clin. Neurophysiol..

[6] Sharma P., Seeck M., Beniczky S. (2019). Accuracy of Interictal and Ictal Electric and Magnetic Source Imaging: A Systematic Review and Meta-Analysis. Front. Neurol..

[7] Brodbeck V., Spinelli L., Lascano A.M., Wissmeier M., Vargas M.I., Vulliemoz S., Pollo C., Schaller K., Michel C.M., Seeck M. (2011). Electroencephalographic source imaging: A prospective study of 152 operated epileptic patients. Brain.

[8] Mouthaan B.E., Rados M., Boon P., Carrette E., Diehl B., Jung J., Kimiskidis V., Kobulashvili T., Kuchukhidze G., Larsson P.G. (2019). Diagnostic accuracy of interictal source imaging in presurgical epilepsy evaluation: A systematic review from the E-PILEPSY consortium. Clin. Neurophysiol..

[9] Abdallah C., Maillard L.G., Rikir E., Jonas J., Thiriaux A., Gavaret M., Bartolomei F., Colnat-Coulbois S., Vignal J.P., Koessler L. (2017). Localizing value of electrical source imaging: Frontal lobe, malformations of cortical development and negative MRI related epilepsies are the best candidates. Neuroimage Clin..

[10] Schneider F., Irene Wang Z., Alexopoulos A.V., Almubarak S., Kakisaka Y., Jin K., Nair D., Mosher J.C., Najm I.M., Burgess R.C. (2013). Magnetic source imaging and ictal SPECT in MRI-negative neocortical epilepsies: Additional value and comparison with intracranial EEG. Epilepsia.

[11] Rampp S., Rossler K., Hamer H., Illek M., Buchfelder M., Doerfler A., Pieper T., Hartlieb T., Kudernatsch M., Koelble K. (2021). Dysmorphic neurons as cellular source for phase-amplitude coupling in Focal Cortical Dysplasia Type II. Clin. Neurophysiol..

[12] Heers M., Hirschmann J., Jacobs J., Dumpelmann M., Butz M., von Lehe M., Elger C.E., Schnitzler A., Wellmer J. (2014). Frequency domain beamforming of magnetoencephalographic beta band activity in epilepsy patients with focal cortical dysplasia. Epilepsy Res..

[13] Frauscher B., Bartolomei F., Kobayashi K., Cimbalnik J., van’t Klooster M.A., Rampp S., Otsubo H., Holler Y., Wu J.Y., Asano E. (2017). High-frequency oscillations: The state of clinical research. Epilepsia.

[14] Schonherr M., Stefan H., Hamer H.M., Rossler K., Buchfelder M., Rampp S. (2017). The delta between postoperative seizure freedom and persistence: Automatically detected focal slow waves after epilepsy surgery. Neuroimage Clin..

[15] Vogel S., Kaltenhauser M., Kim C., Muller-Voggel N., Rossler K., Dorfler A., Schwab S., Hamer H., Buchfelder M., Rampp S. (2021). MEG Node Degree Differences in Patients with Focal Epilepsy vs. Controls-Influence of Experimental Conditions. Brain Sci..

[16] Tzourio-Mazoyer N., Landeau B., Papathanassiou D., Crivello F., Etard O., Delcroix N., Mazoyer B., Joliot M. (2002). Automated anatomical labeling of activations in SPM using a macroscopic anatomical parcellation of the MNI MRI single-subject brain. Neuroimage.

[17] Oostenveld R., Fries P., Maris E., Schoffelen J.M. (2011). FieldTrip: Open source software for advanced analysis of MEG, EEG, and invasive electrophysiological data. Comput. Intell. Neurosci..

[18] Nolte G. (2003). The magnetic lead field theorem in the quasi-static approximation and its use for magnetoencephalography forward calculation in realistic volume conductors. Phys. Med. Biol..

[19] Jung T.-P., Makeig S., Bell A.J., Sejnowski T.J. (1998). Independent Component Analysis of Electroencephalographic and Event-Related Potential Data. Central Auditory Processing and Neural Modeling.

[20] Nolte G., Bai O., Wheaton L., Mari Z., Vorbach S., Hallett M. (2004). Identifying true brain interaction from EEG data using the imaginary part of coherency. Clin. Neurophysiol..

[21] Yekutieli D., Benjamini Y. (1999). Resampling-based false discovery rate controlling multiple test procedures for correlated test statistics. J. Stat. Plan. Inference.

[22] Xu N., Shan W., Qi J., Wu J., Wang Q. (2021). Presurgical Evaluation of Epilepsy Using Resting-State MEG Functional Connectivity. Front. Hum. Neurosci..

[23] Li Hegner Y., Marquetand J., Elshahabi A., Klamer S., Lerche H., Braun C., Focke N.K. (2018). Increased Functional MEG Connectivity as a Hallmark of MRI-Negative Focal and Generalized Epilepsy. Brain Topogr..

[24] Niso G., Carrasco S., Gudin M., Maestu F., Del-Pozo F., Pereda E. (2015). What graph theory actually tells us about resting state interictal MEG epileptic activity. Neuroimage Clin..

[25] Pourmotabbed H., Wheless J.W., Babajani-Feremi A. (2020). Lateralization of epilepsy using intra-hemispheric brain networks based on resting-state MEG data. Hum. Brain Mapp..

[26] Routley B., Shaw A., Muthukumaraswamy S.D., Singh K.D., Hamandi K. (2020). Juvenile myoclonic epilepsy shows increased posterior theta, and reduced sensorimotor beta resting connectivity. Epilepsy Res..

[27] Krishnan B., Vlachos I., Wang Z.I., Mosher J., Najm I., Burgess R., Iasemidis L., Alexopoulos A.V. (2015). Epileptic focus localization based on resting state interictal MEG recordings is feasible irrespective of the presence or absence of spikes. Clin. Neurophysiol..

[28] Aydin Ü., Pellegrino G., Ali O.B.K.B., Abdallah C., Dubeau F., Lina J.-M., Kobayashi E., Grova C. (2020). Magnetoencephalography resting state connectivity patterns as indicatives of surgical outcome in epilepsy patients. J. Neural Eng..

[29] Ramaraju S., Wang Y., Sinha N., McEvoy A.W., Miserocchi A., de Tisi J., Duncan J.S., Rugg-Gunn F., Taylor P.N. (2020). Removal of Interictal MEG-Derived Network Hubs Is Associated With Postoperative Seizure Freedom. Front. Neurol..

[30] Englot D.J., Hinkley L.B., Kort N.S., Imber B.S., Mizuiri D., Honma S.M., Findlay A.M., Garrett C., Cheung P.L., Mantle M. (2015). Global and regional functional connectivity maps of neural oscillations in focal epilepsy. Brain.

[31] Fujiwara H., Kadis D.S., Greiner H.M., Holland K.D., Arya R., Aungaroon G., Fong S.L., Arthur T.M., Kremer K.M., Lin N. (2022). Clinical validation of magnetoencephalography network analysis for presurgical epilepsy evaluation. Clin. Neurophysiol..

[32] Nissen I.A., van Klink N.E., Zijlmans M., Stam C.J., Hillebrand A. (2016). Brain areas with epileptic high frequency oscillations are functionally isolated in MEG virtual electrode networks. Clin. Neurophysiol..

[33] Vespa S., Baroumand A.G., Ferrao Santos S., Vrielynck P., De Tourtchaninoff M., Feys O., Strobbe G., Raftopoulos C., Van Mierlo P., El Tahry R. (2020). Ictal EEG source imaging and connectivity to localize the seizure onset zone in extratemporal lobe epilepsy. Seizure.

[34] He B.J. (2014). Scale-free brain activity: Past, present, and future. Trends Cogn. Sci..

[35] Buzsáki G., Wang X.-J. (2012). Mechanisms of Gamma Oscillations. Annu. Rev. Neurosci..

[36] Sherman M.A., Lee S., Law R., Haegens S., Thorn C.A., Hämäläinen M.S., Moore C.I., Jones S.R. (2016). Neural mechanisms of transient neocortical beta rhythms: Converging evidence from humans, computational modeling, monkeys, and mice. Proc. Natl. Acad. Sci. USA.

[37] Van Diepen R.M., Foxe J.J., Mazaheri A. (2019). The functional role of alpha-band activity in attentional processing: The current zeitgeist and future outlook. Curr. Opin. Psychol..

[38] Isaacson J.S., Scanziani M. (2011). How inhibition shapes cortical activity. Neuron.

[39] Kaltenhauser M., Scheler G., Rampp S., Paulini A., Stefan H. (2007). Spatial intralobar correlation of spike and slow wave activity localisations in focal epilepsies: A MEG analysis. Neuroimage.

[40] De Stefano P., Carboni M., Marquis R., Spinelli L., Seeck M., Vulliemoz S. (2022). Increased delta power as a scalp marker of epileptic activity: A simultaneous scalp and intracranial electroencephalography study. Eur. J. Neurol..

[41] Duez L., Tankisi H., Hansen P.O., Sidenius P., Sabers A., Pinborg L.H., Fabricius M., Rasonyi G., Rubboli G., Pedersen B. (2019). Electromagnetic source imaging in presurgical workup of patients with epilepsy: A prospective study. Neurology.

[42] Baroumand A.G., van Mierlo P., Strobbe G., Pinborg L.H., Fabricius M., Rubboli G., Leffers A.M., Uldall P., Jespersen B., Brennum J. (2018). Automated EEG source imaging: A retrospective, blinded clinical validation study. Clin. Neurophysiol..

