# Supplementary material for: MEG Node Degree for Focus Localization: Comparison with Invasive EEG
Source: Biomedicines. 2023 Feb 2;11(2):438. doi: 10.3390/biomedicines11020438 (PMC9953213; doi:10.3390/biomedicines11020438)

Supplementary Figure S1. Full node degree distributions without thresholding for example case shown in figure 1. For delta, parcellated distribution is shown, as used for the statistical analysis.

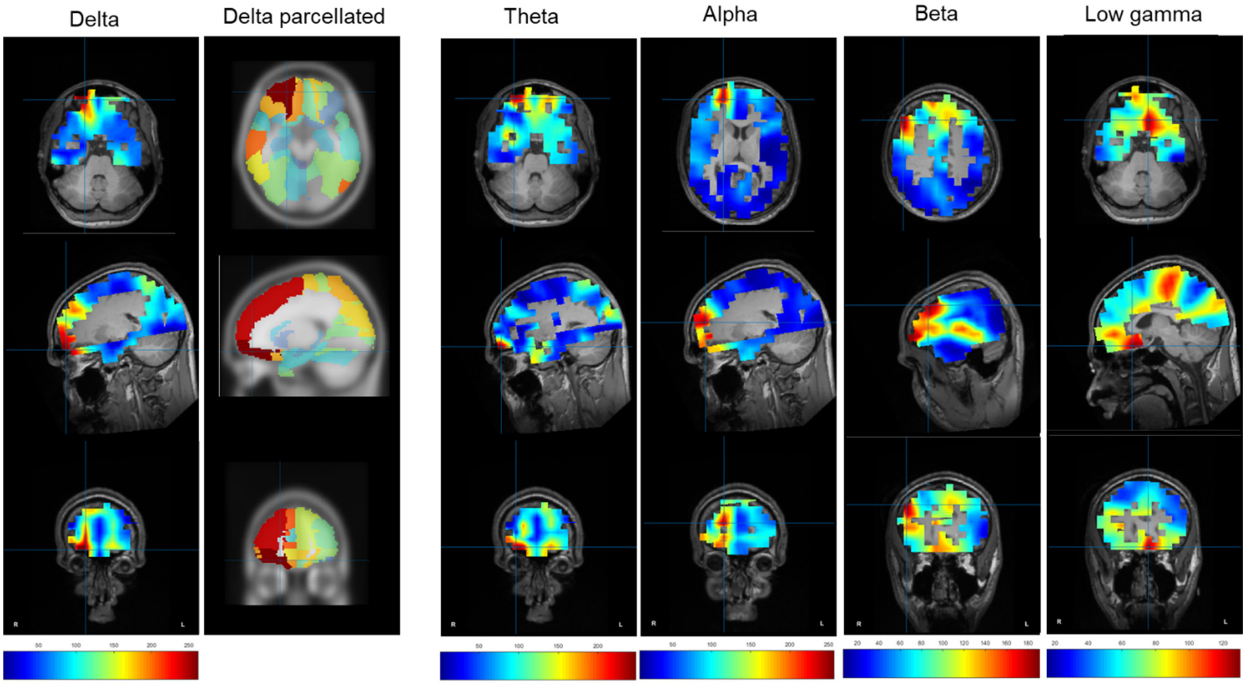

Supplement: Supplementary file 1 [file biomedicines-11-00438-s001.zip › biomedicines-2183458-supplementary.pdf]
